# Exploration of Neural Activity under Cognitive Reappraisal Using Simultaneous EEG-fMRI Data and Kernel Canonical Correlation Analysis

**DOI:** 10.1155/2018/3018356

**Published:** 2018-07-02

**Authors:** Biao Yang, Jinmeng Cao, Tiantong Zhou, Li Dong, Ling Zou, Jianbo Xiang

**Affiliations:** ^1^School of Information Science and Engineering, Changzhou University, Changzhou, Jiangsu 213164, China; ^2^Changzhou Key Laboratory of Biomedical Information Technology, Changzhou, Jiangsu 213164, China; ^3^School of Life Science and Technology, University of Electronic Science and Technology of China, Chengdu, Sichuan 610054, China; ^4^Changzhou No. 2 People's Hospital Affiliated with Nanjing Medical University, Changzhou, Jiangsu 213164, China

## Abstract

**Background:**

Neural activity under cognitive reappraisal can be more accurately investigated using simultaneous EEG- (electroencephalography) fMRI (functional magnetic resonance imaging) than using EEG or fMRI only. Complementary spatiotemporal information can be found from simultaneous EEG-fMRI data to study brain function.

**Method:**

An effective EEG-fMRI fusion framework is proposed in this work. EEG-fMRI data is simultaneously sampled on fifteen visually stimulated healthy adult participants. Net-station toolbox and empirical mode decomposition are employed for EEG denoising. Sparse spectral clustering is used to construct fMRI masks that are used to constrain fMRI activated regions. A kernel-based canonical correlation analysis is utilized to fuse nonlinear EEG-fMRI data.

**Results:**

The experimental results show a distinct late positive potential (LPP, latency 200-700ms) from the correlated EEG components that are reconstructed from nonlinear EEG-fMRI data. Peak value of LPP under reappraisal state is smaller than that under negative state, however, larger than that under neutral state. For correlated fMRI components, obvious activation can be observed in cerebral regions, e.g., the amygdala, temporal lobe, cingulate gyrus, hippocampus, and frontal lobe. Meanwhile, in these regions, activated intensity under reappraisal state is obviously smaller than that under negative state and larger than that under neutral state.

**Conclusions:**

The proposed EEG-fMRI fusion approach provides an effective way to study the neural activities of cognitive reappraisal with high spatiotemporal resolution. It is also suitable for other neuroimaging technologies using simultaneous EEG-fMRI data.

## 1. Introduction

Emotional regulation is known as a unique ability of human beings to control experience and expression of their emotions. It has been the focus of many fields (e.g., cognitive neuroscience, clinical medicine, and sociology) due to its importance to human mental health [1, 2]. Two well-established emotional regulation strategies are widely applied to control emotional experiences, including expressive suppression and cognitive reappraisal. The former is a way of response modulation whereby individual voluntarily inhibits emotional expressive behavior [3]. However, according to the catharsis model, emotions are supposed to “pile up” if not expressed [4]. Hence, expressive suppression may enhance emotional experience which harms mental health. Cognitive reappraisal, on the other hand, is an approach to change the way people think about a potentially emotion eliciting condition to decrease the emotional influence [5]. For instance, one's representative reaction to a scene of a person shooting at another one may be decreased by imaging the scene as a film scene. On the contrary, the reaction may be enhanced by imaging the person is shot by his/her close relative. By utilizing cognitive reappraisal, discomfort to events (e.g., sick, horror, and self-abasement) can be alleviated at an early stage. Despite recent studies show that cognitive reappraisal is correlated to facial frown muscle activities [6], heart rate, and skin conductance [3], studying the essence of cognitive reappraisal is still urgent.

Recently, several neuroimaging technologies (e.g., EEG (electroencephalography) and fMRI (functional magnetic resonance imaging)) are utilized to explore the essence of cognitive reappraisal. Submillisecond temporal resolution of EEG makes it suitable to explore the subtle temporal dynamics of neural activity, which is expressed by electric potential fluctuations spread to the scalp. Event Related Potential (ERP) is widely used to study the characteristics of EEG signals under different emotional states due to its high temporal resolution. An essential component of ERP, Late Positive Potential (LPP), is found to indicate the ability of cognitive reappraisal using emotional regulation. The facilitated processing of emotional stimuli is indicated by the LPP as a central-parietal slow positive deflection in the ERP. The amplitude of LPP turns out to be increased for emotionally eliciting compared with neutral stimuli, beginning with approximately 200ms after stimulus onset and continuing several seconds [7]. Meanwhile, it is susceptible to spontaneous emotional regulation. Hence, a decrease of LPP amplitude can be found when participants are asked to distract attention from the pictures which may arouse unpleasant emotion via cognitive reappraisal [8]. Moreover, LPP reduction can also be found from positive emotional regulation by cognitive reappraisal [9, 10]. However, emotional eliciting sources are hard to locate due to the poor spatial resolution of EEG. FMRI is another widely used technology to study the brain function. It can localize both superficial and deep sources of activity with mm-scale spatial resolution via detecting the variations of blood oxygenation level-dependent (BOLD). Cerebral regions which participate in emotional regulation can be found via fMRI due to its high spatial resolution. Recent fMRI researches show that voluntary reappraisal can influence modulated neural activities in the amygdala [11, 12]. It also indicates that the employment of cognitive reappraisal influences the neural activities in the dorsal parts of the anterior cingulate cortex, the ventromedial prefrontal cortex, and the dorsolateral prefrontal cortex [13]. However, the low resolution temporal variations of these regions are not suitable for studying the neural activity under cognitive reappraisal.

To resolve the abovementioned insufficient of monomodality neuroimaging technology, simultaneous EEG-fMRI fusion is utilized to study the neural activity under cognitive reappraisal due to its high spatiotemporal resolution. In general, there are mainly three approaches for simultaneous EEG-fMRI fusion, including fMRI aided EEG analysis, EEG aided fMRI analysis, and symmetric EEG-fMRI analysis. For fMRI aided EEG analysis, fMRI information with high spatial resolution is used to support the inverse issue of EEG source reconstruction. Kyathanahally* et al*. proposed a framework to invest decision-making in the brain using simultaneous EEG-fMRI data [14]. Thinh* et al*. developed a novel multimodal EEG-fMRI fusion approach by employing the most probable fMRI spatial subsets to guide EEG source localization in a time-variant fashion [15]. For EEG informed fMRI analysis, EEG features (e.g., ERP amplitude, the power spectrum, and epileptic) are used to forecast the BOLD changes in fMRI. Liu* et al*. proposed a general linear model (GLM) model for EEG-fMRI fusion. The fusion results indicate that the intraparietal sulcus and frontal executive areas are the primary sources of biasing influences on task-related visual cortex, whereas task-unrelated default mode network and sensorimotor cortex are suppressive during visual attention [16]. Ahmad* et al*. developed a framework to recognize different visual brain activity patterns using simultaneous EEG-fMRI data. A GLM model was utilized for EEG-fMRI fusion and the results were further classified into different patterns by multilayer perceptron [17]. For symmetric EEG-fMRI analysis, both data are jointly processed by a generative model or changed into a common feature/data space. Yu* et al*. developed a framework to construct multimodal brain graphs using EEG-fMRI data which were simultaneously sampled during eyes open and eyes closed resting states [18]. FMRI data were decomposed into independent components with associated time courses by group independent component analysis (ICA) and EEG time series were segmented into spectral power time courses by superposed average of five frequency bands (alpha, theta, beta, delta, and low gamma). However, ICA assumes that all sources are independent. This strong assumption restricts the power of ICA fusion approach in exploring the underlying sources. Canonical correlation analysis (CCA) was employed by Correa* et al*. to fuse simultaneous EEG-fMRI data with weak assumption [19]. Dong* et al*. also proposed a CCA based EEG-fMRI fusion approach to study familial cortical myoclonic tremor and epilepsy [20]. The proposed local multimodal serial analysis was specifically designed to handle the change of hemodynamic response functions (HRFs).

Despite the widely developed approaches to analyze EEG-fMRI data, there is still no method that focuses on two challenging issues of simultaneous EEG-fMRI fusion; one is to handle the mutual interference between EEG and fMRI, and the other is to handle the nonlinearity of EEG-fMRI data. Aiming to resolve these challenges, we propose an effective fusion framework based on CCA. Empirical mode decomposition (EMD) is used to increase SNR of EEG data that is polluted by MR scanning. FMRI masks are constructed and are used to eliminate unwanted fMRI components that are correlated with wanted EEG components. RBF kernel is embedded into the CCA framework to handle the nonlinearity of EEG-fMRI data. Participants are shown with visual stimuli paradigm. Based on previous researches that study EEG and fMRI, respectively [7], we expect (1) the correlated ERP components and fMRI activated regions related to cognitive reappraisal can be simultaneously be extracted from the EEG-fMRI data, and (2) LPPs under three emotional states can be observed from the correlated ERP components and amplitudes of different LPPs coincided with the existed studies, and (3) the correlated fMRI activated regions coincided with the previous found regions (e.g., amygdala, dorsomedial PFC, dorsolateral prefrontal cortex (PFC), anterior cingulate cortex, and orbitofrontal cortex). There are mainly two contributions of our work: (1) providing an effective framework for simultaneous EEG-fMRI fusion and (2) exploring the neural activity under cognitive reappraisal in high spatiotemporal resolution.

## 2. Materials and Methods

### 2.1. The EEG-fMRI Fusion Framework

**Figure 1:**
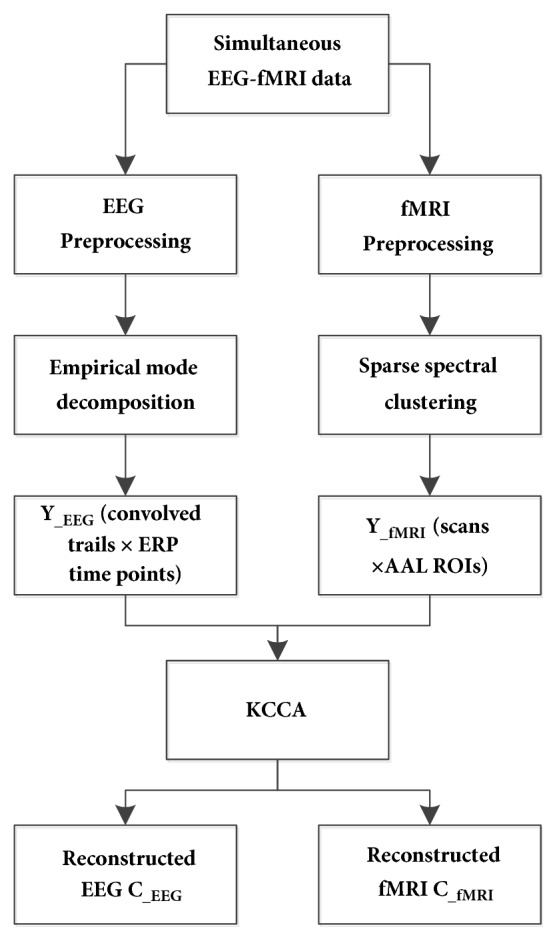
Pipeline of the proposed EEG-fMRI fusion approach.

The framework of the fusion approach is demonstrated in Figure 1. Simultaneous EEG-fMRI data is preprocessed, respectively. EMD is further used to eliminate noise of EEG data. Sparse spectral clustering (SSC) is employed to construct fMRI masks that indicate the emotion-related cerebral regions. EEG feature to be fused is defined as Y__EEG_ (convolved trails × ERP time points), which are obtained by convolving the ERP values at different time points with a standard HRF. On the other hand, fMRI feature to be fused is defined as Y__fMRI_ (scans ×AAL ROIs), which are obtained by calculating mean values in anatomical automatic labeling (AAL) cerebral regions under the constraints of fMRI masks. Then, Y__EEG_ and Y__fMRI_ are fused using a kernel-based CCA (KCCA) framework. EEG and fMRI components (C__EEG_ and C__fMRI_) are finally reconstructed based on the selected correlated components.

### 2.2. Subjects

A total of 15 healthy adults, 5 females and 10 males, aged from 19 to 24 years (M (mean value) =23, SD (standard deviation) =1.48), are recruited from Changzhou University to implement the experiments.

Participants have regular or corrected regular vision without history of neurological, medical, or psychiatric disorders. They have been tested for psychological profile to discard some comorbid issues as depression or psychiatric symptoms that can affect emotional evaluation. All participants provide written informed consent to be part of the experiment, which is approved by the local ethics committee (Changzhou University, Changzhou, China). Each subject receives 42-minute fMRI scan (structure: 5 min, resting state: 5 min, and task state: 32 min).

### 2.3. Paradigm

**Figure 2:**
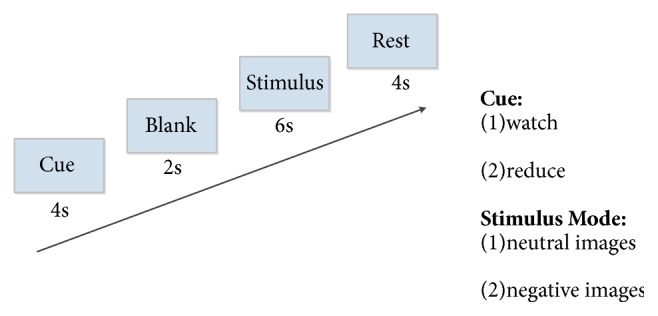
Illustration of the visual stimuli paradigm.

The visual stimuli paradigm [21] is implemented in a block fMRI design as shown in Figure 2. The entire experiment for one participant contains 120 trials, including 4 circulations in which 30 trials are implemented. Three conditions, including watching neutral images (e.g., buildings, neutral faces, and food), watching negative images (e.g., sadness, disasters, and violence), and watching negative images with cognitive reappraisal, are randomly implemented in 40 trials, respectively. All the images used are chosen from the international affective picture gallery. The arousal for neutral images is M (mean) = 2.91 and SD (standard deviation) = 1.93; meanwhile, for negative images it is M = 5.71 and SD = 2.61. Procedure of a single trial can last at most 16 seconds as proposed in [22]. Initially, cue word “reduce” or “watch” is shown on the screen for 4 seconds in the cue period. After a 2-second blank period, the stimulus period will last for 6 seconds. At this period, neutral and negative images will randomly appear with cue word “watch”, while only negative images will appear with cue word “reduce”. Notably, cognitive reappraisal will be used if the cue word “reduce” appears. Finally, the rest period will last for 4 seconds.

### 2.4. Simultaneous EEG-fMRI Acquisition

EEG acquisition system of EGI company (Eugene, the USA) is used in the experiment. EEG is sampled continuously at 1000Hz. An amplifier is placed inside the MR scanner room. Subjects are fitted with an electrode cap containing 64 electrodes with Cz as online reference. Later, the data are referenced to zero by reference electrode standardization technique [23]. It is recently confirmed being close to the idea of zero reference [24, 25]. Impedances are kept low below 50kΩ. The helium pump is turned off during experiments to avoid related artifacts.

**Figure 3:**
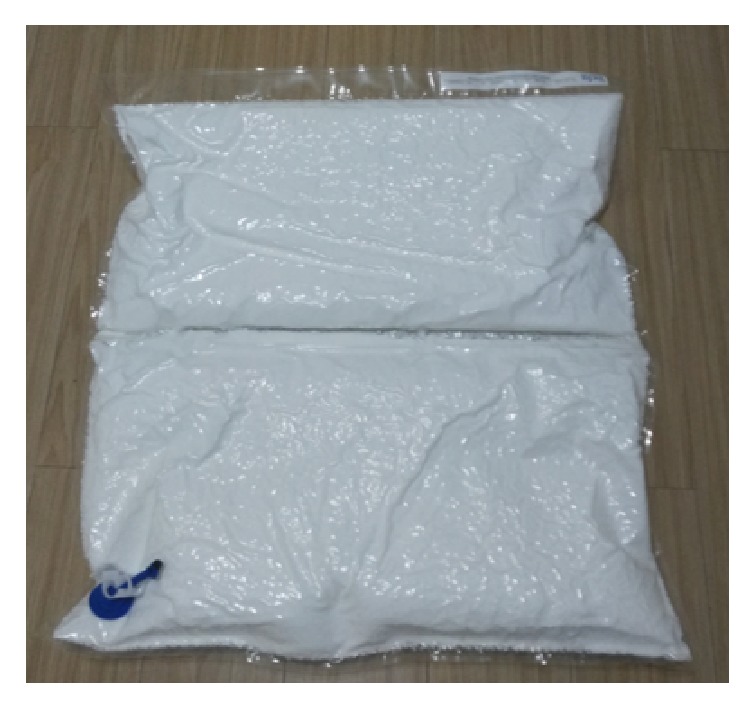
The foam pads used to prevent head movement.

Functional imaging data are sampled with 3-Tesla superconducting type nuclear magnetic resonance imaging system of Philips Company. Single excitation gradient echoes planar sequence is utilized to acquire functional images. After a whole paradigm finished, 960 BOLD sensitive echo planar images (EPI) are gathered during four sessions. EPI volumes are aligned with the anterior-posterior commissural line. It contains 24 axial slices with 4mm thickness including flip angle: 90 degree; TR (repetition time): 2s; TE (echo time): 35ms; FOV (field of view): 230mm*∗*182mm; matrix: 96×74. Subjects are mandated to lie in the MRI scanning room, staying awake, and blinking as little as possible. The foam pads (Figure 3) are used to prevent head movement.

### 2.5. EEG Data Processing

Processing of EEG data contains two parts, one is denoising and the other is extracting EEG feature. In consideration of the influence caused by MR scanning, denoising is achieved through two steps: traditional denoising using net-station toolbox and further increasing SNR using EMD.

For traditional denosing, noises such as gradient artifact, ECG, and power interference are eliminated as follows: (1) Gradient artifact is removed by template elimination method. The gradient artifact template is constructed in a weighted average mean by labeling the timing that fMRI triggered EEG. Then, an average artifact subtraction method is utilized to eliminate the gradient artifact. (2) Band-pass filtering is employed with the band 0.01-40Hz. (3) Optimal basis set approach is used to eliminate ballistocardiogram artifacts caused by the heartbeat. (4) The EEG data are segmented into different fragments based on the stimulus time point. Each fragment ranges from 200ms before stimulus and 1500ms after it. (5) Artifacts such as head movements and blinking are detected in all fragments of all electrodes. The electrode with artifacts is labeled as bad electrode. (6) The bad electrode is replaced by the average of its 3 surrounding electrodes. (7) The first 200ms of each fragment is used for baseline correction.

After traditional denoising, EMD is employed to further increase SNR of EEG data that is affected by MR scanning [26]. EMD tries to find functions which form a complete and nearly orthogonal basis of the original signal. These functions are termed as Intrinsic Mode Functions (IMFs). Then, increasing SNR can be achieved through removing IMFs that are taken as disturbance. Details of increasing SNR through EMD can be found in our former work [27].

After denoising, emotion-related ERP extracted from EEG is used to study the neural activity under cognitive reappraisal [7]. Amplitudes of ERP (extracted from Poz channel) at different time points are termed as EEG feature. At each time point, the trial-to-trial dynamics are convolved with a standard HRF to coincide with fMRI (5 volumes in each trial) due to the BOLD delay. We restrict the analysis to 900ms (225 uniform and consecutive time points) after stimulus onset because the most emotion-related components in the EEG are considered to appear during the first 200-700ms after stimulus onset. Finally, the dimension of the extracted EEG feature is 600 (convolved trails) × 225 (ERP time points).

### 2.6. FMRI Data Processing

FMRI data are processed using reference electrode standardization technique and statistical parametric mapping (SPM) to correct slice time and exclude head motion. Then the data are normalized and further registered to the Montreal Neurological Institute (MNI) space. Finally, a Gaussian filter (full-width at half-maximum of 8 mm) is used for smoothing filtering and only five fMRI activation regions (three in stimulus period and two in rest period) after stimulus presentation are selected in each trial. Each fMRI activation region is represented by its mean values in different AAL ROIs [28]. Finally, the dimension of the extracted fMRI feature is 600 (scans) × 90 (AAL ROIs).

Notably, some fMRI regions irrelevant to emotion processing are also activated. These undesired activation regions should be removed to guarantee the accuracy of EEG-fMRI fusion. Otherwise, they may correlate with the wanted EEG components. In this work, an fMRI mask is constructed through spatiotemporal clustering of all fMRI activation. SSC is used to cluster the fMRI activation because SSC is insensitive to the number of features and, thus, can avoid dimension disaster [29]. Then, there is no undesired fMRI activation in the fMRI mask because undesired activation mostly sustain for a short period in certain cerebral regions. Finally, for each row of the fMRI feature, an “and” operation with fMRI mask will be performed to restrain the influence of undesired fMRI activation.

### 2.7. Simultaneous EEG-fMRI Data Fusion Using KCCA

CCA searches for a pair of linear transformations of the variable set in the manner of one for each. It is commonly used for symmetric EEG-fMRI analysis. Given two data X (Y__EEG_) and Y (Y__fMRI_), their generative models are given by

(1) X=AXCXY=AYCY

where* A*_*X*_ and* A*_*Y*_ are canonical variate matrices and* C*_*X*_ and* C*_*Y*_ are associated EEG and fMRI components. Let* a*_*Xk*_ and* a*_*Yk*_ represent the* k*^*th*^ column of* A*_*X*_ and* A*_*Y*_ (the* k*^*th*^ pair of canonical variate); then their relational degree is defined as

(2) ρk=aXkTSXYaYkaXkTSXXaXk×aYkTSYYaYk

(3) S=SX,Y=SXXSXYSYXSYY

where* ρ*_*k*_ indicates the relational degree of the* k*^*th*^ pair of associated components. The total covariance matrix* S* is represented as a block matrix. The within-sets covariance matrices are* S*_*XX*_ and* S*_*YY*_. The between-sets covariance matrices are *S*_XY_= S_YX_^T^. Then, those associated components whose relational degrees are larger than a given threshold (0.55) are used to reconstruct the wanted EEG component ${\hat{C}}_{X}$ and fMRI component ${\hat{C}}_{Y}$, which are defined as follows:

(4) C^X=A^XTA^X−1A^XTXC^Y=A^YTA^Y−1A^YTY

where ${\hat{A}}_{X}$ and ${\hat{A}}_{Y}$ only contain the selected pairs of canonical variate. Details of solving a CCA problem can be referred to [19].

However, CCA cannot process nonlinear data. Thus, kernel is used to resolve such problem through mapping data into a high dimensional feature space. A kernel* κ* for all X, Y *∈* R is defined as follows:

(5) $$\begin{array}{c} \kappa \left(\;X,Y\;\right)=\langle \;\varphi \left(\;X\;\right),\varphi \left(\;Y\;\right)\;\rangle \end{array}$$

where* φ* is a mapping from the original data space* R* to a new feature space* F* (*φ*: R->F). Great flexibility can be achieved by applying different kernels such as linear kernel, Gaussian kernel, and RBF. Based on kernel, the directions* a*_*Xk*_ and* a*_*Yk*_ can be represented as follows:

(6) aXk=XαaYk=Yβ

where *α* and *β* indicate the transformations from original data to their canonical variate. Then, (2) can be represented as follows:

(7) $$\begin{array}{c} \rho_{k}=\frac{\alpha^{\prime }X^{\prime }XY^{\prime }Y\beta^{\prime }}{\sqrt{\alpha^{\prime }X^{\prime }XX^{\prime }X\alpha •\beta^{\prime }Y^{\prime }YY^{\prime }Y\beta }} \end{array}$$

Notable, linear transformations X'X and Y'Y cannot process nonlinear data very well. Hence, RBF kernel is used to replace the linear transformations due to its superiority in processing nonlinear data. Then, (2) can be rewritten as follows:

(8) $$\begin{array}{c} \rho_{k}=\frac{\alpha^{\prime }K_{X}K_{Y}\beta }{\sqrt{\alpha^{\prime }{K_{X}}^{2}\alpha •\beta^{\prime }{K_{Y}}^{2}\beta }} \end{array}$$

where* K*_*X*_ and* K*_*Y*_ represent the RBF kernel matrices. Relational degrees calculated using (8) is more suitable to nonlinear EEG-fMRI data than that calculated using (2).

## 3. Experimental Results

### 3.1. Comparisons between KCCA and CCA

This work focuses on the highly correlated components between EEG temporal evolution and fMRI spatial activation. Ninety correlated components are obtained using KCCA fusion.  Table 1 demonstrates the correlated components whose relational degrees are larger than 0.55. As shown in the table, there are six pairs of correlated components under neutral and reappraisal states, and seven pairs of correlated components under negative state. Our former work using CCA fusion is used as comparison [27]. It is obvious that relational degrees obtained using KCCA is larger than that obtained using CCA.

**Figure 4:**
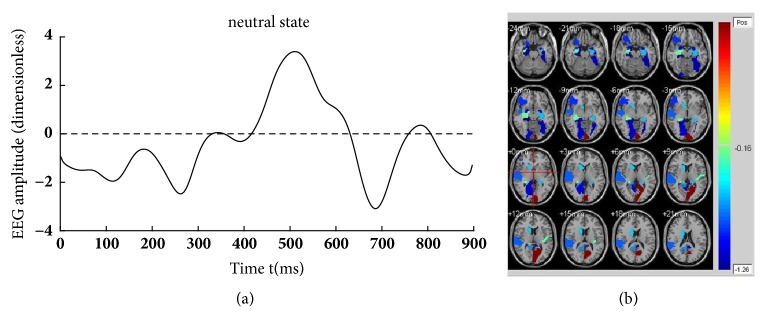
Fifteen subjects' superposed average result of correlated EEG-fMRI under neutral state. (a) Correlated EEG component and (b) correlated fMRI activation.

**Figure 5:**
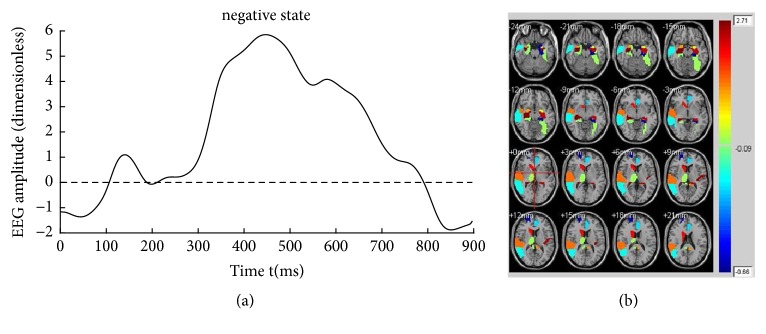
Fifteen subjects' superposed average result of correlated EEG-fMRI under negative state. (a) Correlated EEG component and (b) correlated fMRI activation.

**Figure 6:**
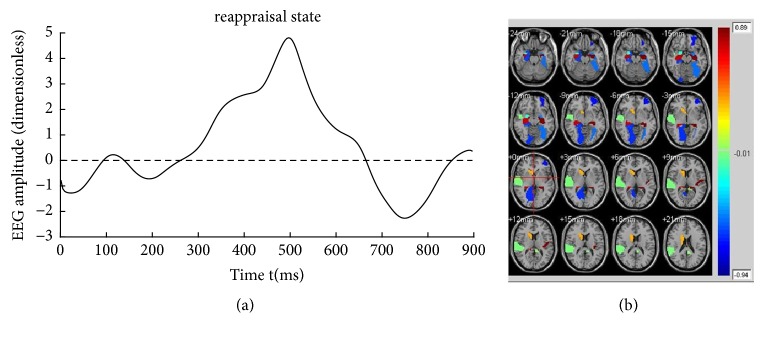
Fifteen subjects' superposed average result of correlated EEG-fMRI under reappraisal state. (a) Correlated EEG component and (b) correlated fMRI activation.

Figures 4–6 illustrate the fifteen subjects' superposed average results of the correlated EEG-fMRI components. Correlated components whose relational degrees are larger than 0.55 are used for superposed average. For each figure, subfigure (a) indicates the superposed average result of correlated EEG component extracted from Poz electrode. Furthermore, x-axis represents time (ms) and y-axis represents normalized amplitude (dimensionless). Subfigure (b) illustrates the correlated fMRI activation under the same state, while the color-bar indicates the normalized activated intensity. Then, neural activities caused by the same stimuli can be observed in both high temporal (correlated EEG component) and spatial resolutions (correlated fMRI activation).

**Figure 7:**
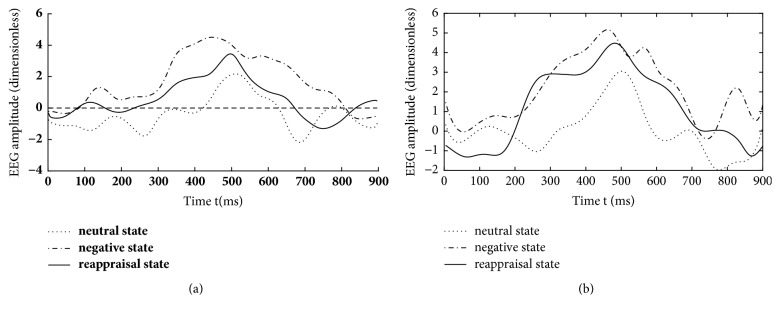
Fifteen subjects' superposed average results of EEG correlation components under three emotional states using (a) KCCA fusion and (b) CCA fusion.

Aside from the differences in relational degrees, differences in EEG components are also evaluated between CCA [27] and KCCA.  Figure 7(a) illustrates the fifteen subjects' superposed average results of reconstructed EEG components that are calculated by KCCA fusion. All the EEG components are extracted from Poz electrode. Obvious differences can be observed among their LPP components. The amplitude of LPP component under reappraisal state is smaller than that under negative state and is obviously larger than that under neutral state.  Figure 7(b) illustrates the fifteen subjects' superposed average results of reconstructed EEG components that are calculated by CCA fusion. Similar results can be observed. However, amplitudes of LPP component under negative state and that under reappraisal state are more or less intersecting at the reported emotion arousing period (200-700ms by [7]) as illustrated in Figure 7(b). The intersection may be caused by nonlinearity of simultaneous EEG-fMRI data.

A quantitative comparison is performed on the average correlated EEG components of fifteen subjects under three emotional states. 225 samples are uniformly sampled from 700ms EEG component and their amplitudes are used as input. F-test is used for evaluation and different emotional states are used as the factors of ANOVA. The result shows distinct differences in EEG components of different emotional states. The mean of the differences (MOD) between conditions under negative and neutral states is 23, with F (1, 224) = 262.65(P < 0.01). The MOD between conditions under reappraisal and negative states is 11, with F (1, 224) = 70.49(P < 0.01). The MOD between conditions under reappraisal and neutral states is 13, when F (1, 224) = 83.04(P < 0.01). Obviously, the quantitative result is confirmed to the result of Figure 7.

### 3.2. Comparisons between KCCA and GLM

Comparisons between KCCA and GLM are performed to verify the superiority of symmetric EEG-fMRI analysis in studying the neural activities of cognitive reappraisal.  Table 2 demonstrates fifteen subjects' superposed average results of correlated fMRI activation under three emotional states using KCCA. Intensities of fMRI activation are measured by the Z-score values in different AAL ROIs. A big Z-score value indicates a strong fMRI activation. Notably, only AAL ROIs whose Z-score values are larger than 0 (a negative Z-score value in certain AAL ROI indicates that this ROI is irrelevant to emotion processing) are preserved in this table. Meanwhile, no EEG component is evaluated because GLM is mainly used for analyzing fMRI activation.  Table 3 demonstrates fifteen subjects' superposed average results of correlated fMRI activation under three emotional states using GLM. Differences between KCCA and GLM exist in both activated regions and intensities. Discussions of their differences will be given in Section 4 in detail.

### 3.3. Evaluation of the fMRI Masks

**Figure 8:**
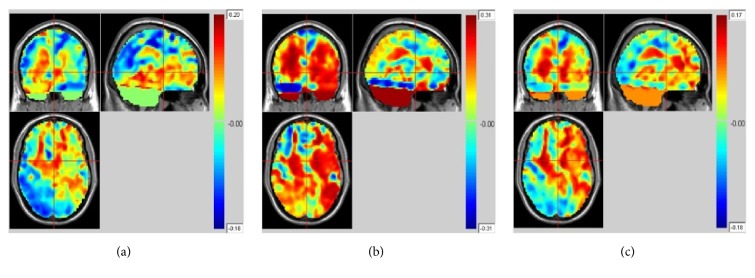
fMRI clustering results of all subjects (a) under neutral state; (b) under negative state; and (c) under reappraisal state (color-bar indicates the activated intensity).

FMRI masks are used to restrain the activated fMRI regions due to their ability to eliminate the regions uncorrelated to emotion processing. The clustering results of all subjects under three emotional states are illustrated in Figure 8. As shown in the figure, activated regions under neutral state are the smallest while activated regions under negative state are the biggest.

**Figure 9:**
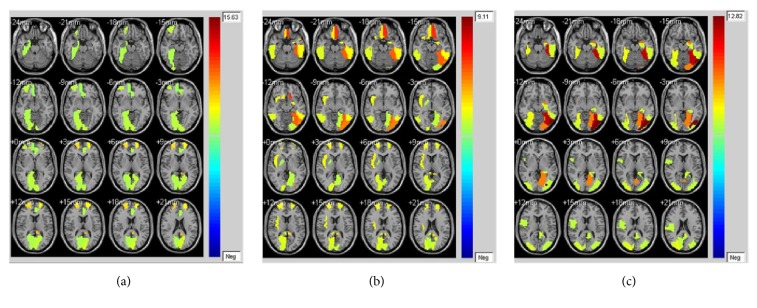
Fifteen subjects' superposed average results of correlated fMRI activation using the proposed method without fMRI masks (a) under negative state, (b) under reappraisal state, and (c) under neutral state.

KCCA fusion without fMRI masks is performed to evaluate the effectiveness of fMRI masks. For fMRI, fifteen subjects' superposed average results of correlated fMRI activation obtained through KCCA but without fMRI masks are illustrated in Figure 9. Correlated fMRI activation varies a lot due to whether fMRI masks are used, especially under negative and reappraisal states. For example, there is obvious activation in cerebral regions such as hippocampus, amygdala, and temporal lobe that are directly related to emotion processing in Figure 5(b). However, no activation can be found in these cerebral regions in Figure 9(a). Same phenomena can be observed in Figures 6(b) and 9(b). There is no activation in emotion-related cerebral regions such as hippocampus and temporal lobe in Figure 9(b), while obvious activation can be observed in these regions in Figure 6(b). There is no obvious difference in activated fMRI regions between Figures 4(b) and 9(c) because fMRI masks do not focus on cerebral regions unrelated to emotion processing.

**Figure 10:**
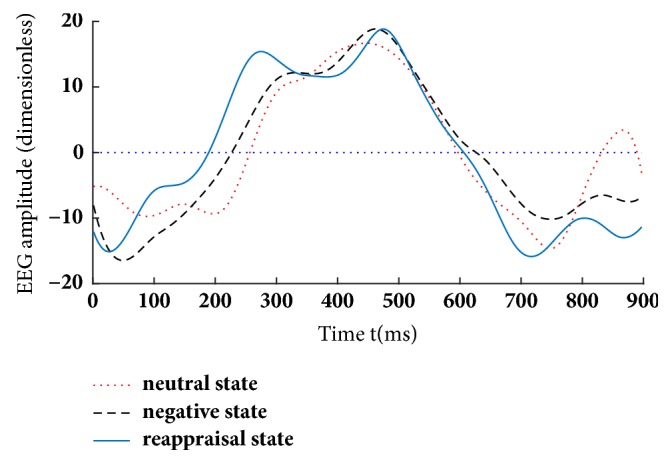
Fifteen subjects' superposed average results of EEG correlated components under three emotional states using the proposed method but without clustering mask.

For EEG, fifteen subjects' superposed average results of EEG correlated components under three emotional states but without clustering mask are shown in Figure 10. Compared with the results in Figure 7, no obvious decrease can be observed in ERP amplitude from negative state to reappraisal state. Meanwhile, EEG evolutions under different emotional states are hard to separate.

## 4. Discussion and Conclusion

The aim of cognitive reappraisal is to regulate human experience under negative emotion such as depression, fear, and disappointment. Simultaneous EEG-fMRI analysis is used to study the neural activity under cognitive reappraisal due to its complementarity in both spatial and temporal domains. In this work, these neural activities are studied using a KCCA fusion framework. Meanwhile, EMD is used to further increase SNR of EEG data that is sampled under MR scanning. FMRI masks are calculated using SSC and are used to eliminate the activation unrelated to emotion processing. With all these processing, both EEG and fMRI components can be reconstructed based on the selected correlated components (Figures 4, 5, and 6). Results of these figures are very important to study the mechanism of cognitive reappraisal that is useful for human to regulate his/her emotion. For spatial analysis, activation in emotion-related cerebral regions (e.g., amygdala, hippocampus, and temporal lobe) under reappraisal state is obviously weaker than that under negative state through introducing the cognitive reappraisal strategy. It reveals that negative emotion can be effectively restricted in emotion-related cerebral regions after applying cognitive reappraisal strategy. For temporal analysis, obvious differences can be observed among different LPP components which are considered to be highly correlated to emotion processing. Peak value of LPP component under reappraisal state is smaller than that under negative state, and obviously larger than that under neutral state. Both the shrunken fMRI activated regions and decreased peak value of LPP component verify the assumptions that negative emotions, e.g., sorrow, fear, and disappointment, can be restrained by using cognitive reappraisal.

Effectiveness of kernel strategy can be observed through the comparisons between KCCA and CCA. CCA fusion is widely used for symmetric EEG-fMRI analysis. However, nonlinearity of the EEG-fMRI data may decrease the fusion accuracy. Thus, we improve the CCA fusion with a kernel strategy. It is not very novel but is effective. KCCA fusion is specially designed to process nonlinear EEG-fMRI data. As shown in Table 1, relational degrees of correlated components derived using KCCA fusion are mainly larger than that derived by CCA fusion. Notably, a larger relational degree indicates a stronger relationship between two components. Thus, the results in Table 1 may indicate the superiority of KCCA fusion to traditional CCA fusion.

The superiority of KCCA fusion to CCA fusion can be also observed from the reconstructed EEG and fMRI components. For fMRI that concentrates on spatial activation, no obvious activation can be observed in hippocampus which is emotion-related under negative or reappraisal states using CCA fusion. It may be caused by the fact that CCA cannot process nonlinear EEG-fMRI data. However, obvious activation can be observed in these regions under the same emotional states using KCCA fusion. It reveals the ability of KCCA in mining effective fMRI activation from nonlinear EEG-fMRI data. For EEG that concentrates on temporal evolutions, amplitude of LPP component under reappraisal state is obviously weaker than that under negative state at the same period using KCCA fusion. The decrease in amplitude indicates the ability of cognitive reappraisal to restrain sorrowful emotion, as pointed out by [4]. However, no obvious decrease can be observed in amplitude of LPP component from negative state to reappraisal state using CCA fusion. Thus, the larger relational degrees, the more fMRI activation, and the obvious decrease in amplitude of LPP component between negative and reappraisal states reveal the superiority of KCCA fusion to CCA fusion. Such superiority is obtained due to the effect of kernel strategy in processing nonlinear EEG-fMRI data.

The superiority of symmetric EEG-fMRI analysis to EEG informed fMRI analysis can be observed through the comparisons between KCCA and GLM (Tables 2 and 3). Only fMRI activation is compared because GLM cannot be used to study the EEG evolutions. Then, for KCCA (Table 2), obvious activation can be observed under negative state in cerebral regions such as the temporal lobe, the hippocampus, the amygdala, and the cingulate gyrus. Meanwhile, activation can be observed under reappraisal state in cerebral regions such as the amygdala, the temporal lobe, the cingulate gyrus, the hippocampus, and the frontal lobe. These activation regions indicate the important role of these cerebral regions in emotional regulation. In the perspective of activated intensity (Z-score), activation in cerebral regions under reappraisal state, especially the regions (e.g., the amygdala, the hippocampus, and the temporal lobe) directly related to emotion processing, is obviously weaker than activation in those regions under negative state through using cognitive reappraisal. Activation in these cerebral regions under neutral state is much weaker than activation in the same regions under the other two states. All these results are basically consistent with the conclusions proposed by [28]. Compared with the fusion results obtained using GLM (Table 3), two results can be concluded: (1) by utilizing KCCA approach, more regions are found to be activated under negative and reappraisal states, and (2) activated intensities of these regions calculated using KCCA fusion are larger than those calculated using GLM fusion. Both results indicate the superiority of KCCA fusion (symmetric EEG-fMRI analysis) in studying the neural activity of cognitive reappraisal.

As a special preprocessing, fMRI masks are useful due to the assumption that strong fMRI activation uncorrelated with emotion processing may be correlated with EEG components, thus leading to omitting the fMRI activation which we are truly interested in. As shown in Figures 4(b), 5(b), and 6(b), obvious fMRI activation can be observed in emotion-related cerebral regions such as the hippocampus and the temporal lobe under negative and reappraisal states. However, no activation can be observed in these regions if fMRI masks are not used as preprocessing. Meanwhile, obvious decrease can be observed in EEG amplitude from negative state to reappraisal state using our fusion approach (Figure 7(a)), while little decrease can be observed under the same condition without fMRI masks (Figure 10).

Based on the above discussions, our fusion approach may provide a fine solution for analyzing simultaneous EEG-fMRI data in high resolution spatiotemporal domains. It can synchronously tell when and where the neural activities related to certain tasks such as cognitive reappraisal occur. It may also provide a useful technological means for fusion-based cerebral area positioning, ERP-induction time determination, and brain imaging feature extraction in the area of brain-human interface. Our fusion approach can be also used in paradigms which can cause LPP with further study on cognitive researches and clinical trials.

There are still some limitations in the proposed fusion approach: (1) for the data to be fused, activation in AAL ROIs is employed instead of original fMRI voxels, aiming at reducing computational complexity. As a result, one cannot study the activation of reconstructed fMRI components at voxel level. (2) Prior knowledge is necessary for our KCCA fusion approach. It is hard to choose suitable parameters that are significant for satisfactory fusion results. There is also no certain criterion in determining the threshold of relational degrees. (3) The number of enrolled subjects is far from enough; thus, the evaluations may lack persuasion. Our future work focuses on implementing EEG-fMRI fusion at voxel level instead of AAL ROIs. Thus, the spatial resolution of reconstructed fMRI components can be greatly boosted.

## Figures and Tables

**Table 1:** Correlated components of EEG-fMRI with high relational degrees (> 0.55) under three emotional states.

correlated components	relational degrees (KCCA approach / CCA approach)		
	neutral state	negative state	reappraisal state
component 1	**0.951** / 0. 944	**0.971 **/ 0.966	**0.932 **/ 0.913
component 2	**0.892** / 0.888	**0.952** / 0.947	**0.834 **/ 0.801
component 3	0.833 /** 0.841**	0.863 / **0.877**	**0.805 **/ 0.731
component 4	**0.765 **/ 0.741	**0.821 **/ 0.816	0.704 / **0.716**
component 5	0.643 / **0.681**	0.753 / **0.765**	**0.613 **/ 0.606
component 6	**0.586** / N/A	**0.712** / 0.660	**0.551** / 0.537
component 7	N/A / N/A	**0.605 **/ 0.584	N/A / N/A

**Table 2:** Fifteen subjects' superposed average results of correlated fMRI activations under neutral state, negative state, and reappraisal state (using KCCA).

under neutral state		under negative state		under reappraisal state	
AAL ROIs (No)	Z-score	AAL ROIs (No)	Z-score	AAL ROIs (No)	Z-score
Calcarine_L (43)	0.355	Hippocampus_R (38)	2.711	Heschl_L (79)	0.887
Hippocampus_R (38)	0.208	Heschl_L (79)	2.485	Hippocampus_L (37)	0.870
Heschl_L (79)	0.178	Hippocampus_L (37)	2.317	Hippocampus_R (38)	0.776
Caudate_R (72)	0.068	Caudate_R (72)	1.990	Caudate_R (72)	0.550
N / A	N / A	Temporal_Sup_R (82)	1.383	Amygdala_ R (42)	0.043
N / A	N / A	Cingulum_Post_L (35)	1.249	Temporal_Sup_R (82)	0.038
N / A	N / A	Amygdala_R (42)	1.233	Amygdala_L (41)	0.031
N / A	N / A	Cingulum_Mid_R (34)	0.672	Cingulum_Post_L (35)	0.006
N / A	N / A	Cingulum_Mid_L (33)	0.627	N / A	N / A
N / A	N / A	Amygdala_L (41)	0.567	N / A	N / A
N / A	N / A	Fusiform_L (55)	0.223	N / A	N / A
N / A	N / A	Thalamus_R (78)	0.220	N / A	N / A
N / A	N / A	Cingulum_Post_R (36)	0.121	N / A)	N / A
N / A	N / A	ParaHippocampal_R (40)	0.090	N / A	N / A

**Table 3:** Fifteen subjects' superposed average results of correlated fMRI activations under neutral state, negative state, and reappraisal state (using GLM).

under neutral state		under negative state		under reappraisal state	
AAL ROIs (No)	Z-score	AAL ROIs (No)	Z-score	AAL ROIs (No)	Z-score
Parietal_Sup_L (59)	0.516	Heschl_L (79)	1.587	Parietal_Inf_R (62)	0.887
Paracentral_Lobule_R (70)	0.366	Parietal_Sup_L (59)	1.466	Parietal_Sup_L (59)	0.870
Parietal_Sup_R (60)	0.159	Parietal_Sup_R (60)	1.039	Occipital_Mid_R (52)	0.776
Occipital_Mid_L (51)	0.020	Precuneus_L (67)	0.922	ParaHippocampal_L (39)	0.350
N / A	N / A	Paracentral_Lobule_L (69)	0.790	Angular_R (66)	0.006
N / A	N / A	Paracentral_Lobule_R (70)	0.725	N / A	N / A
N / A	N / A	Occipital_Mid_R (52)	0.569	N / A	N / A
N / A	N / A	Occipital_Mid_L (51)	0.507	N / A	N / A
N / A	N / A	Occipital_Sup_R (50)	0.478	N / A	N / A
N / A	N / A	Parietal_Inf_L (61)	0.292	N / A	N / A
N / A	N / A	Temporal_Pole_Mid_L (87)	0.159	N / A	N / A
N / A	N / A	SupraMarginal_L (63)	0.126	N / A	N / A
N / A	N / A	Precuneus_R (68)	0.116	N / A	N / A
N / A	N / A	SupraMarginal_R (64)	0.114	N / A	N / A
N / A	N / A	Heschl_R (80)	0.108	N / A	N / A
N / A	N / A	Cingulum_Mid_L (33)	0.084	N / A	N / A
N / A	N / A	ParaHippocampal_L (39)	0.080	N / A	N / A
N / A	N / A	Cingulum_Ant_L (31)	0.022	N / A	N / A

## Data Availability

The data used to support the findings of this study are available from the corresponding author upon request.
